# Factors Associated with In-Hospital Mortality Among Adults Receiving ECMO: A Nationwide Cohort Study (2011–2020)

**DOI:** 10.3390/jcm15051770

**Published:** 2026-02-26

**Authors:** Hsiao-En Tsai, Wen-Chun Tsai, Shu-Chuan Weng, Yih-Sharng Chen, Shoei-Shen Wang, Chia-Pang Shih

**Affiliations:** 1 Graduate Institute of Clinical Medicine, College of Medicine, National Taiwan University, Taipei 100, Taiwan; smilen@gmail.com (H.-E.T.); yschen1234@gmail.com (Y.-S.C.); wangp@ntu.edu.tw (S.-S.W.); 2 Division of Cardiovascular Surgery, Critical Care Medicine Center, National Taiwan University Hospital, Hsin-Chu Branch, Hsinchu 300, Taiwan; 3 Department of Nursing, En Chu Kong Hospital, New Taipei City 237, Taiwan; 10682@km.eck.org.tw; 4 Department of Nursing, National Yang Ming Chiao Tung University, Taipei 112, Taiwan; 5 Department of Gerontological Health Care, Yuanpei University of Medical Technology, Hsinchu 300, Taiwan; scweng303@mail.ypu.edu.tw; 6 Division of Cardiovascular Surgery, National Taiwan University Hospital, Taipei 100, Taiwan; 7 Department of Management, Chung Shan Medical University Hospital, Taichung 402, Taiwan; 8 Department of Healthcare Management, Yuanpei University of Medical Technology, Hsinchu 300, Taiwan

**Keywords:** extracorporeal membrane oxygenation, in-hospital mortality, nationwide cohort, care trajectory, real-world data

## Abstract

**Background/Objectives:** Extracorporeal membrane oxygenation (ECMO) use has increased worldwide, yet in-hospital mortality among adult recipients remains substantial. Large-scale evidence examining patient- and treatment-related factors associated with mortality in real-world settings is still limited. This study aimed to quantify in-hospital mortality and identify factors associated with mortality among adults receiving ECMO using a nationwide cohort in Taiwan. **Methods:** We conducted a retrospective nationwide cohort study using Taiwan’s National Health Insurance Research Database, including adults (≥18 years) who received ECMO during hospitalization between 2011 and 2020. ECMO indication groups were defined using ICD-9-CM (before 2016) and ICD-10-CM (2016 onward) codes and further classified into four mutually exclusive categories. Multivariable logistic regression was used to examine factors associated with in-hospital mortality. **Results:** Among 15,151 adults treated with ECMO, 9657 (63.7%) died during hospitalization. In multivariable analyses, higher odds of in-hospital mortality were associated with older age, higher comorbidity burden (Charlson Comorbidity Index ≥3), and use of multiple ECMO machines (≥2). Compared with patients without cardiopulmonary indications, those classified as cardiogenic shock alone or combined respiratory failure and cardiogenic shock had lower adjusted odds of in-hospital mortality. Longer hospital length of stay was inversely associated with in-hospital mortality, reflecting differing care trajectories among ECMO recipients. **Conclusions:** In this nationwide real-world cohort of adult ECMO recipients, in-hospital mortality was high, and mortality risk was associated with patient age, comorbidity burden, ECMO treatment complexity, and diagnosis-based indication classification. These findings provide population-level insight into mortality patterns and may inform risk communication and system-level planning for ECMO care.

## 1. Introduction

Over the past two decades, extracorporeal membrane oxygenation (ECMO) has become an established life-support modality in intensive care, providing temporary circulatory and respiratory support for patients with severe cardiac or pulmonary failure [1]. The two primary configurations of ECMO are veno-arterial (VA) ECMO, which is mainly used for circulatory support, and veno-venous (VV) ECMO, which is primarily applied for respiratory failure [2,3]. VA ECMO is most commonly indicated for conditions such as cardiogenic shock and post-cardiotomy cardiac failure and may be used in selected cases of sepsis-associated circulatory collapse, whereas VV ECMO is typically utilized for severe acute respiratory distress syndrome and refractory bacterial or viral pneumonia.

The duration of ECMO support may range from several hours to several weeks, depending on the underlying condition and treatment goals [4]. ECMO has been widely applied as a bridge-to-transplant or bridge-to-recovery strategy, allowing critically ill patients additional time for organ recovery or definitive treatment. However, despite increasing clinical experience and the development of practice guidelines, there remains substantial heterogeneity in patient selection, treatment duration, and criteria for defining successful ECMO outcomes, and decisions regarding ECMO initiation often rely on multidisciplinary clinical judgment [5].

Recent studies have reported an increasing volume of ECMO use and gradual improvements in survival outcomes [5,6]. Nevertheless, in-hospital mortality among ECMO-treated patients remains high. Reported mortality rates range from approximately 35% to 60% in patients with cardiogenic shock and from 47% to 57% in those with refractory respiratory failure, despite advances in supportive care [7,8,9,10,11]. Given the expanding utilization of ECMO and its substantial resource demands, there is a continued need for population-level evidence to clarify factors associated with in-hospital mortality. Therefore, this study aimed to examine patient-, treatment-, and hospital-level factors associated with in-hospital mortality among adults receiving ECMO, using nationwide data from Taiwan’s National Health Insurance Research Database between 2011 and 2020.

## 2. Materials and Methods

### 2.1. Study Population and Data Source

This nationwide retrospective cohort study utilized data from the Taiwan National Health Insurance Research Database (NHIRD) for the period between 2011 and 2020. We identified 15,151 adult patients (aged ≥ 18 years) who received extracorporeal membrane oxygenation (ECMO) during hospitalization. Ethics approval for this study was obtained from the Institutional Review Board of National Taiwan University Hospital HsinChu Branch (No. 110-073-E).

### 2.2. Definition of ECMO Indications

To identify the clinical conditions prompting ECMO initiation, ECMO indication groups were operationalized using ICD-9-CM diagnosis codes for admissions prior to 2016 and ICD-10-CM diagnosis codes for admissions from 2016 onward. Diagnosis codes recorded across the first five diagnosis fields of the index hospitalization were screened. Patients could be classified into one or more indication groups if any qualifying diagnosis code was present, reflecting the frequent coexistence of multiple critical conditions in ECMO recipients. The predefined indication groups included respiratory failure, cardiogenic shock/cardiac indications, post-cardiotomy, sepsis, cardiopulmonary failure (pulmonary embolism or pulmonary vascular/pulmonary heart disease), trauma/hypothermia/drowning, and heart transplant. Detailed ICD-9-CM and ICD-10-CM code sets are provided in Appendix A Table A1.

Given the substantial clinical overlap among critically ill ECMO recipients, we further constructed a secondary disease classification to approximate the predominant ECMO indication for analytical purposes. This classification was based specifically on the presence or absence of respiratory failure and cardiogenic shock codes and consisted of four mutually exclusive categories: (1) isolated respiratory failure, (2) isolated cardiogenic shock, (3) combined respiratory failure and cardiogenic shock, and (4) other indications. Importantly, this secondary classification was independent of other indication groups, such as cardiopulmonary failure or sepsis, which were retained as separate variables in the primary indication analysis.

### 2.3. Covariates and Confounders

We controlled for a comprehensive set of potential confounders, including patient demographics (age and sex), socioeconomic status (proxied by insurance premium levels), and comorbidity burden quantified using the Charlson Comorbidity Index. Treatment-related factors, including the use of multiple ECMO machines (≥2 vs. 1), cannula replacement during ECMO support, and hospital length of stay (LOS), were also evaluated. To minimize residual confounding and account for variations in healthcare delivery patterns, institutional characteristics—including hospital level and geographic region—were incorporated into the multivariable regression models as adjustment factors.

### 2.4. Statistical Analysis

The normality of continuous variables was assessed using the Shapiro–Wilk test. Categorical variables are presented as frequencies and percentages, while continuous variables are summarized as means with standard deviations or medians with interquartile ranges, as appropriate. Comparisons between survivors and non-survivors were conducted using the chi-square test or Fisher’s exact test for categorical variables, and the two-sample *t*-test or Mann–Whitney U test for continuous variables.

Logistic regression analyses were performed to identify factors associated with in-hospital mortality among ECMO recipients. Variables with a *p*-value < 0.10 in univariable analyses were entered into the multivariable model, with results reported as adjusted odds ratios (ORs) and 95% confidence intervals (CIs). In supplementary analyses, LOS was examined as a categorized variable for descriptive comparisons with discharge outcomes. To ensure the robustness of our findings, a temporal sensitivity analysis was performed by repeating the analyses in the 2020 cohort. All statistical analyses were conducted using SAS version 9.4 (SAS Institute Inc., Cary, NC, USA), and a two-sided *p*-value < 0.05 was considered statistically significant.

## 3. Results

### 3.1. Comparison of Characteristics Between Survivors and Non-Survivors

#### 3.1.1. Demographic Characteristics

We included 15,151 patients in the study. Among them, 5494 patients (36.3%) survived to hospital discharge, whereas 9657 patients (63.7%) died during hospitalization. The mean age of the overall cohort was 57.0 ± 15.3 years. Patients who died in hospital were older than those who survived (59.0 ± 14.9 vs. 54.0 ± 15.5 years, *p* < 0.0001). Male patients accounted for 10,617 cases (70.1%) of the study population. The proportion of male patients did not differ significantly between the survival and death groups (*p* = 0.30) (Table 1).

#### 3.1.2. Clinical Characteristics

No statistically significant difference was observed in the number of ECMO machines used between survivors and non-survivors (*p* = 0.06). The majority of patients received a single ECMO machine during hospitalization (93.3%), and the proportion of patients requiring multiple machines was similar between the two groups (6.2% among survivors vs. 7.0% among non-survivors).

Cannula replacement during ECMO support occurred more frequently among survivors than among non-survivors (30.1% vs. 28.2%, *p* = 0.01).

Significant differences in the distributions of admission diagnoses were identified between the survival and death groups (Table 1). Respiratory failure, cardiopulmonary failure, trauma/hypothermia/drowning, and heart transplant were more common among survivors, whereas cardiogenic shock and sepsis were more prevalent among non-survivors.

To account for overlapping diagnoses, admission diagnoses were further categorized into four mutually exclusive groups: isolated respiratory failure, isolated cardiogenic shock, combined respiratory failure and cardiogenic shock, and neither condition. Patients with isolated respiratory failure or combined respiratory and cardiogenic failure accounted for a larger proportion of survivors, while those with isolated cardiogenic shock or neither condition were more frequently observed among non-survivors (*p* < 0.0001).

Comorbidity burden, assessed using the Charlson Comorbidity Index (CCI), differed significantly between outcome groups (*p* < 0.0001). Patients with no comorbidities or a CCI score of 1–2 were more frequently observed among survivors, whereas patients with a CCI score of 3 or higher accounted for a larger proportion of in-hospital deaths (Table 1).

#### 3.1.3. Hospital Course

With respect to hospital course variables, the length of stay differed markedly between outcome groups. The median length of stay among patients who died during hospitalization was 9 days (interquartile range [IQR], 20 days), whereas the median length of stay among survivors was 35 days (IQR, 43 days) (*p* < 0.0001). No significant difference in socioeconomic status was observed between the survival and death groups (*p* = 0.34) (Table 1).

Regarding hospital-level characteristics, most ECMO recipients were treated at medical centers (*n* = 9553, 63.1%). The distribution of hospital accreditation level differed significantly between survivors and non-survivors (*p* = 0.001). Among survivors, 3567 (64.9%) were treated at medical centers, 1870 (34.0%) at regional hospitals, and 57 (1.0%) at district hospitals. Among non-survivors, 5986 (62.0%) were treated at medical centers, 3547 (36.7%) at regional hospitals, and 124 (1.3%) at district hospitals. Only a small proportion of ECMO recipients received care in district hospitals (*n* = 181, 1.2%) (Table 1).

### 3.2. Factors Associated with In-Hospital Mortality

We performed logistic regression analyses to identify factors associated with in-hospital mortality among patients receiving ECMO. In univariate analyses, age, cannula replacement, diagnosis classification, Charlson Comorbidity Index (CCI) category, length of stay, hospital level, and geographic region were significantly associated with in-hospital mortality (Table 2).

In terms of diagnosis classification, patients with respiratory failure alone or with both respiratory failure and cardiogenic shock had lower odds of in-hospital mortality compared to those with neither condition in univariate analyses. For comorbidity burden, patients without comorbidities had lower odds of in-hospital mortality, while those with a CCI of 3 or higher had increased odds. Longer length of stay was also associated with reduced odds of in-hospital mortality. Socioeconomic status was not significantly associated with in-hospital mortality.

Variables with *p*-values < 0.10 in univariable analyses were entered into the multivariable logistic regression model. Accordingly, age, diagnosis classification, Charlson Comorbidity Index (CCI) category, cannula replacement, length of stay, and hospital accreditation level were included. Sex and socioeconomic status were not included because they did not meet the predefined screening criterion in univariable analyses (*p* < 0.10). In the multivariable analysis, age, use of ≥2 ECMO machines, diagnosis classification, CCI category, and length of stay remained independently associated with in-hospital mortality. The associations for cannula replacement and hospital accreditation level were attenuated and were no longer statistically significant after adjustment (Table 2).

### 3.3. Length of Stay as an Indicator of Care Trajectory

We examined length of stay (LOS) as an indicator of care trajectory, applying a 25-day reference point in supplementary descriptive analyses. Among patients treated between 2011 and 2019 (*n* = 13,206), we observed that prolonged hospitalization (LOS ≥ 25 days) occurred more frequently among those who survived to discharge, whereas shorter hospital stays predominated among patients who experienced early adverse outcomes (Table 3).

We repeated the comparable descriptive comparison in the 2020 cohort as a temporal sensitivity analysis and observed a similar pattern (Table 3).

Collectively, these results indicate that survival following ECMO is more commonly associated with resource-intensive, prolonged care trajectories rather than brief clinical courses.

## 4. Discussion

ECMO has served as a form of temporary cardiopulmonary support for more than five decades, and its utilization has increased substantially worldwide in recent years [12,13]. In this nationwide retrospective cohort of over 15,000 adult ECMO recipients in Taiwan, we observed a high in-hospital mortality rate of 63.7%, underscoring the extreme illness severity of this population and the clinical importance of identifying factors associated with adverse outcomes. Notably, the observed lower adjusted mortality among patients classified as cardiogenic shock or combined respiratory and cardiogenic failure should be interpreted cautiously, as these associations likely reflect patient selection and system-level care patterns rather than intrinsic differences in disease severity.

Using multivariable analyses, we identified several patient-, treatment-, and system-level factors associated with in-hospital mortality. The multivariable model demonstrated good overall discrimination for mortality; however, the primary aim of this study was to examine associations at the population level rather than to develop or validate a prognostic scoring system.

Consistent with prior registry-based studies, advanced age and higher comorbidity burden were strongly associated with increased in-hospital mortality [14]. Each additional year of age was associated with a 2% increase in mortality risk, and patients with a high comorbidity burden (CCI ≥ 3) had a 43% higher adjusted odds of death, whereas the absence of comorbidity was associated with modestly lower mortality. While the influence of baseline health status and physiological reserve is a general consideration in critical care, it holds particular significance in the context of ECMO therapy. As a highly resource-intensive, last-resort intervention, ECMO outcomes depend not only on acute stabilization but also on the patient’s intrinsic capacity to tolerate prolonged extracorporeal support and subsequent recovery. Our findings demonstrate that a high comorbidity burden (CCI ≥ 3) is strongly associated with increased in-hospital mortality, underscoring the limiting role of reduced physiological reserve in this setting. In patients with substantial baseline comorbidities, diminished reserve may constrain the ability to recover from prolonged critical illness despite successful short-term circulatory or respiratory support. Accordingly, these results highlight the importance of incorporating baseline comorbidity burden into ECMO candidate evaluation, alongside acute illness severity, to better contextualize expected clinical trajectories.

Beyond traditional patient-level risk factors, our analysis demonstrated that ECMO indication groups derived from admission diagnoses were independently associated with in-hospital mortality. In adjusted models, respiratory failure was no longer associated with reduced mortality, whereas patients classified as having cardiogenic shock or combined respiratory and cardiogenic failure exhibited significantly lower adjusted mortality compared with patients without cardiopulmonary indications.

Although these findings appear counterintuitive when compared with traditional prognostic frameworks for ECMO, they likely reflect real-world utilization patterns rather than intrinsic differences in disease lethality. Previous studies have consistently reported high mortality among patients receiving ECMO for cardiogenic shock [7,15], yet these analyses largely focused on physiologic severity at cannulation rather than clinical selection processes. In contemporary practice, patients with cardiogenic shock are more likely to be managed within standardized care pathways, receive earlier ECMO initiation, and undergo closer hemodynamic monitoring, which may mitigate adverse outcomes despite severe underlying pathology. Conversely, the survival benefit in the cardiogenic shock group may be tied to the prevalence of standardized VA-ECMO care pathways in Taiwan’s major medical centers. It is important to note that diagnosis-based ECMO indication groups derived from administrative data inherently encompass heterogeneous underlying etiologies. For example, the respiratory failure category includes a broad spectrum of conditions ranging from acute respiratory distress syndrome and aspiration-related hypoxemia to drowning and other causes of acute respiratory collapse, which may differ in baseline severity, reversibility, and response to extracorporeal support. Similarly, cardiogenic shock may arise from diverse etiologies with varying clinical trajectories. Since the NHIRD relies on administrative ICD codes, our analysis cannot distinguish these specific underlying mechanisms or their individual physiological trajectories. Accordingly, the observed associations should be interpreted as reflecting population-level patterns of ECMO utilization and outcomes rather than prognostic differences attributable to specific disease mechanisms. Clinicians should therefore remain mindful of this etiology-driven variability when applying these findings to individual bedside decision-making. Similar observations regarding the influence of institutional processes and center volume on ECMO outcomes have been reported in large registry-based studies. In this study, the use of multiple ECMO machines was independently associated with higher in-hospital mortality. Although patients who survive longer may have a greater opportunity to experience circuit-related events requiring exchange, we interpret the need for multiple machines primarily as a marker of greater underlying disease severity and treatment complexity. Patients requiring multiple circuits often represent those with refractory circulatory failure or circuit-related complications, such as thrombosis or oxygenator failure, necessitating system escalation or replacement. Accordingly, the use of multiple ECMO machines should be viewed as a proxy for a complicated clinical course rather than a direct effect of prolonged survival. Because the NHIRD does not capture detailed procedural information, we were unable to distinguish whether the use of multiple ECMO machines reflected sequential circuit exchanges, oxygenator replacement, re-cannulation, or other technical complications. Accordingly, this variable represents a heterogeneous marker of treatment complexity rather than a specific failure mechanism.

Regarding procedural factors, cannula replacement was observed more frequently among survivors in univariate analysis. Cannula or circuit-related interventions during ECMO support have been reported as common complications in large registry studies and are generally considered indicators of treatment complexity rather than independent prognostic factors. Accordingly, cannula replacement likely reflects a prolonged and complicated clinical course, and is inherently subject to survivor bias, as patients must survive long enough to encounter cannulation- or circuit-related issues requiring intervention. This interpretation is further supported by the loss of statistical significance after multivariable adjustment, suggesting that underlying illness severity and duration of support, rather than the replacement procedure itself, drive observed outcomes.

The association between LOS and clinical outcomes in this ECMO-treated cohort reflects a distinct treatment trajectory rather than a prognostic determinant. Shorter hospitalization among non-survivors primarily reflects early mortality, indicating rapid clinical deterioration during the initial treatment course in a subset of critically ill patients. In contrast, prolonged hospitalization was more frequently observed among patients who survived to discharge, reflecting extended supportive care and recovery processes following severe critical illness. Accordingly, LOS should be interpreted as a marker of clinical trajectory, with shorter stays indicating early adverse outcomes and longer stays highlighting the resource-intensive nature of care among survivors. Importantly, LOS was not intended to function as a causal risk factor in the regression analysis, but rather as a process-dependent marker reflecting the clinical care trajectory, the endpoint of which is intrinsically defined by the occurrence of discharge or death.

In contrast, patients without clearly defined cardiopulmonary indications may represent a more heterogeneous population, including individuals with complex comorbid conditions, atypical indications, or delayed initiation of extracorporeal support. Importantly, these results should not be interpreted as suggesting that cardiogenic shock or combined cardiopulmonary failure is inherently associated with better prognosis. Rather, diagnosis-based indication groups reflect the characteristics of patients selected for ECMO under contemporary clinical practice and the institutional processes governing ECMO deployment. Similar observations highlighting the influence of center experience and organizational factors on ECMO outcomes have been reported in large registry-based and volume–outcome studies [14,16]. Taken together, our findings underscore both the utility and the inherent limitations of using admission diagnosis-based classifications to approximate ECMO indications in large-scale population-based research.

From a clinical and health-system perspective, our findings suggest that admission diagnosis-based ECMO indication grouping may provide a pragmatic framework for early risk stratification in large healthcare databases. Although such classifications cannot replace physiologic severity assessment, they reflect real-world patient selection and institutional practice patterns that influence outcomes. In resource-intensive therapies such as ECMO, this approach may facilitate benchmarking across hospitals, inform early family counseling, and support system-level planning. Importantly, our results emphasize that ECMO outcomes are shaped not only by patient severity but also by organizational factors governing ECMO deployment.

Several limitations of this study should be acknowledged. First, this was a retrospective observational study based on administrative claims data, which are inherently subject to coding errors and misclassification. Although we used predefined ICD-9-CM and ICD-10-CM code sets to identify ECMO indication groups, diagnosis-based classification may not fully capture clinical severity or physiologic status at the time of ECMO initiation. Moreover, the NHIRD lacks granular laboratory and physiologic data, precluding differentiation of specific disease mechanisms or individual physiologic trajectories within these categories. Accordingly, diagnosis-based associations in this study should be interpreted at the population level rather than as disease-specific prognostic estimates. In addition, variable selection for the multivariable model was guided by univariable screening (*p* < 0.10), a pragmatic approach often adopted in large observational studies to reduce model complexity. Nevertheless, we acknowledge that this strategy may not fully account for all clinically relevant confounders, particularly those that are weakly associated with the outcome in univariate analyses or unavailable in administrative data. As such, residual confounding cannot be excluded, and the observed associations should be interpreted in the context of these modeling constraints.

Second, the lack of granular clinical data on specific ECMO modes (VA vs. VV) in the NHIRD remains an important limitation. Although respiratory failure and cardiogenic shock groups generally correspond to VV and VA configurations, respectively, this administrative proxy cannot account for mode switching or hybrid configurations (e.g., V-A-V). Such misclassification may attenuate differences in outcomes between indication groups, as patients with more complex, dual-organ failure might be simplified into a single category. Furthermore, to the extent that mode transition occurred during the clinical course, the mortality associations we observed might represent a conservative estimate of physiological impact. Future studies integrating clinical registries are essential to validate these diagnosis-based findings against specific cannulation strategies. In addition, the hospital accreditation level was used to characterize the institutional setting. In Taiwan, medical centers account for the majority of ECMO utilization and generally possess greater resources, multidisciplinary teams, and infrastructure to support complex ECMO care. Nevertheless, hospital accreditation remains an imperfect proxy for ECMO-specific case volume, team experience, and organizational maturity. The attenuation of hospital-level effects after multivariable adjustment may therefore reflect residual confounding related to unmeasured differences in ECMO expertise across institutions. Accordingly, hospital-level associations observed in this study should be interpreted cautiously and should not be viewed as direct measures of ECMO center experience. Third, the study period spanned the transition from ICD-9-CM to ICD-10-CM coding, which may have introduced inconsistencies in disease classification despite the use of harmonized code mappings. In addition, unmeasured confounding related to clinical decision-making, patient selection, and institutional practice patterns cannot be fully excluded.

Fourth, analyses were restricted to adult patients who received ECMO, precluding comparisons with non-ECMO populations and limiting inference regarding ECMO candidacy or treatment effectiveness. Therefore, the findings should be interpreted as associations within an ECMO-treated cohort rather than causal effects.

Finally, although the multivariable model demonstrated good discriminatory performance, external validation in other healthcare systems is warranted to assess generalizability beyond Taiwan’s single-payer setting. Nevertheless, the large nationwide cohort and comprehensive follow-up enhance the robustness and representativeness of our findings.

## 5. Conclusions

This nationwide real-world study demonstrates that both patient and institutional factors significantly influence survival outcomes for adults receiving ECMO. The findings offer a comprehensive perspective on mortality patterns, which may enhance risk communication and inform hospital resource planning. ECMO survival remains a resource-intensive process, and optimizing outcomes requires balancing individual patient care with system capacity and hospital workflow considerations.

## Figures and Tables

**Table 1:** Comparison of Demographic and Basic Information Between Surviving and Deceased Patients.

Variables	Overall		Survive		Death		*p*-Value
	*n*	%	*n*	%	*n*	%	
Numbers of patients	15,151		5494	36.3	9657	63.7	
Age, mean (std)	57	(15.3)	54	(15.5)	59	(14.9)	<0.0001 ^1^
Sex, male	10,617	70.1	3822	69.6	6795	70.4	0.30 ^2^
Number of machines							0.06 ^2^
1	14,134	93.3	5153	93.8	8981	93.0	
>=2	1017	6.7	341	6.2	676	7.0	
Replacement of cannulas, yes	4376	28.9	1655	30.1	2721	28.2	0.01 ^2^
Diagnosis							
Respiratory failure	6519	43.0	2687	48.9	3832	39.7	<0.0001 ^2^
Cardiogenic shock	9004	59.4	3180	57.9	5824	60.3	0.003 ^2^
Post-cardiotomy	612	4.0	236	4.3	376	3.9	0.23 ^2^
Sepsis	2956	19.5	854	15.5	2102	21.8	<0.0001^2^
Cardiopulmonary failure	3765	24.8	1503	27.4	2262	23.4	<0.0001 ^2^
Trauma/hypothermia/drowning	757	5.0	314	5.7	443	4.6	0.002 ^2^
Heart transplant	167	1.1	99	1.8	68	0.7	<0.0001 ^2^
None of the above	1025	6.8	371	6.8	654	6.8	0.96 ^2^
Disease classification							<0.0001 ^2^
Respiratory failure	3309	21.8	1361	24.8	1948	20.2	
Cardiogenic shock	5794	38.3	1854	33.8	3940	40.8	
Both conditions	3210	21.2	1326	24.1	1884	19.5	
Neither conditions	2838	18.7	953	17.4	1885	19.5	
CCI classification							<0.0001 ^2^
0	3328	22.0	1428	26.0	1900	19.7	
1–2	6555	43.3	2454	44.7	4101	42.5	
>=3	5268	34.8	1612	29.3	3656	37.9	
Length of stay,							
median (IQR)	17	(33)	35	(43)	9	(20)	<0.0001 ^3^
Social Economic Status							0.34 ^2^
Low	4980	32.9	1765	32.1	3215	33.3	
Middle	8907	58.8	3264	59.4	5643	58.4	
High or unknown	1264	8.3	465	8.5	799	8.3	
Hospital Accreditation							0.001 ^2^
Medical centers	9553	63.1	3567	64.9	5986	62.0	
Regional hospitals	5417	35.8	1870	34.0	3547	36.7	
District Hospitals	181	1.2	57	1.0	124	1.3	

std: standard deviation; IQR: interquartile range; CCI: Charlson Comorbidity Index. ^1^ *p*-value was performed by two sample *t* test. ^2^ *p*-value was performed by chi-square test. ^3^ *p*-value was performed by Mann–Whitney U test.

**Table 2:** Logistic Regression Analysis of Studied Variables Associated with Mortality.

Variables	Univariate				Multivariable			
	OR	95% CI of OR		*p*-Value	OR ^4^	95% CI of OR		*p*-Value
Age	1.02	1.02	1.03	<0.0001	1.02	1.02	1.03	<0.0001
Sex ^1^	1.04	0.97	1.12	0.30				
Number of machines ^2^	1.14	0.99	1.30	0.06	1.32	1.11	1.57	0.002
Replacement of cannulas ^3^	1.10	1.02	1.18	0.01	0.92	0.84	1.01	0.10
Diagnosis classification								
Respiratory failure	0.72	0.65	0.80	<0.0001	0.95	0.84	1.07	0.40
Cardiogenic shock	1.07	0.98	1.18	0.14	0.67	0.60	0.76	<0.0001
Both conditions	0.72	0.65	0.80	<0.0001	0.63	0.56	0.72	<0.0001
Neither condition	1.00				1.00			
CCI classification								
0	0.80	0.73	0.87	<0.0001	0.90	0.82	1.00	0.04
1–2	1.00				1.00			
>=3	1.36	1.26	1.47	<0.0001	1.43	1.31	1.57	<0.0001
Length of stay	0.94	0.94	0.95	<0.0001	0.94	0.94	0.95	<0.0001
Social Economic Status								
Low	1.00							
Middle	0.95	0.88	1.02	0.16				
High	0.94	0.83	1.07	0.37				
Hospital Accreditation								
Medical center	1.00				1.00			
Regional hospital	1.13	1.05	1.21	0.001	1.07	0.98	1.16	0.12
District Hospital	1.30	0.95	1.78	0.11	0.92	0.65	1.30	0.63

CCI: Charlson Comorbidity Index; OR: odds ratio; CI: confidence interval. ^1^ reference = Female; ^2^ reference = Using one ECMO machine; ^3^ reference = Cannula change was performed during ECMO use; ^4^ OR was adjusted by the hospital’s National Health Insurance regional branch.

**Table 3:** Distribution of discharge outcomes by length of stay category.

Length of Stay Category	Survived, *n* (%)	In-Hospital Mortality, *n* (%)
2011–2019		
<25 days	1687 (35.51)	6527 (77.20)
≥25 days	3064 (64.49)	1928 (22.80)
2020		
<25 days	255 (34.32)	914 (76.04)
≥25 days	488 (65.68)	288 (23.96)

Note: Percentages are calculated within each discharge outcome column.

## Data Availability

The data analyzed in this study were obtained from Taiwan’s National Health Insurance Research Database and are not publicly available due to legal and ethical restrictions. Access to the data is subject to approval by the relevant data governance authorities.

## Abbreviations

The following abbreviations are used in this manuscript:

ECMO	Extracorporeal membrane oxygenation
NHIRD	National Health Insurance Research Database
NHI	National Health Insurance
ICD	International Classification of Diseases
CCI	Charlson Comorbidity Index
CI	Confidence interval
OR	Odds ratio
LOS	Length of Stay

## Appendix A

### ICD-9-CM and ICD-10-CM Code Sets for ECMO Indication Groups

This appendix provides the detailed ICD-9-CM and ICD-10-CM diagnosis code sets used to operationalize ECMO indication groups during the index hospitalization.

**Table A1:** ICD-10-CM and ICD-9-CM code sets used to identify ECMO indication groups.

ECMO Indication Group	ICD-10-CM Code Set	ICD-9-CM Code Set
Respiratory failure (RF)	J09–J18, J40–J99	Influenza/pneumonia and related: 487.0, 487.1, 487.8, 073.0, 115.15, 115.95, 481, 482.1, 482.2, 482.30–482.32, 482.39, 482.40–482.41, 482.49, 482.89, 482.9, 483.0–483.1, 484.7–484.8, 485, 486, 514, 517.1; plus ranges 480.0–480.2, 480.8–480.9, 482.81–482.83
Cardiogenic shock/cardiac indications (CS)	I05–I08, I20–I25, I33–I35, I40–I42, I46, I49–I51	Valvular/other cardiac: 393.0–396.3, 396.8–396.9, 394.0–394.2, 394.9, 395.0–395.2, 395.9, 397.0, 397.9; Acute MI: 410.00–410.02, 410.10–410.12, 410.20–410.22, 410.30–410.32, 410.40–410.42, 410.50–410.52, 410.60–410.62, 410.71–410.72, 410.80–410.82, 410.90–410.92; Ischemic heart disease/angina/other: 411.0, 411.1, 411.81, 411.89, 412, 413.0, 413.1, 413.9, 414.00–414.05, 414.10, 414.11, 414.19, 414.8; other: 429.2, 429.5, 429.6, 429.71, 429.79
Postcardiotomy (post-cardiotomy shock/complication)	I97	429.4, 457.0, 997.02, 997.1, 997.91, 998.11, 998.12, 998.2
Sepsis	A40–A41	038.0, 038.11, 038.19, 038.2, 038.3, 038.40–038.44, 038.49, 038.8, 038.9
Cardiopulmonary failure/pulmonary embolism & pulmonary heart disease	I26–I28	415.0, 415.11, 415.19, 416.0, 416.1, 416.8, 416.9, 417.0, 417.1, 417.8, 417.9
Trauma/hypothermia/drowning	Drowning/submersion: W65–W74; Injury codes: S *, T0 *; Hypothermia: T68; Drowning-related: T751	injury/fracture: 733.81–733.82 and extensive injury code lists spanning 800.xx–959.xx, plus V54.8 and V58.89
Lung transplant	Z94.2	V42.6
Heart transplant	Z94.1, T86.2	V42.1, 996.83

Note: Asterisks (*) denote wildcard characters representing all subcodes within the specified ICD-9-CM or ICD-10-CM prefix.
